# Open science and epistemic equity: opportunities and challenges in the contemporary research ecosystem

**DOI:** 10.1186/s13104-025-07608-2

**Published:** 2025-12-11

**Authors:** Robert Farrow

**Affiliations:** Institute of Educational Technology, The Open University (UK), Walton Hall, Kents Hill, Milton Keynes, MK7 6AA UK

## Abstract

The contemporary context for open science is characterised by growing international policy momentum toward more transparent, inclusive, and collaborative scientific practices which build on the transformative potential of openness to enhance research equity, reproducibility, and collaboration. However, the shift from aspiration to implementation remains uneven and contested. Persistent structural challenges (e.g. misaligned incentives, disparities in infrastructure, and uneven access to funding and training) risk reinforcing rather than dismantling existing inequalities. The rapid proliferation of artificial intelligence (AI) technologies, particularly generative AI (GenAI), raises new concerns about epistemic authority, research integrity, and the ownership of knowledge. While AI could accelerate discovery and increase access to scientific information, it may also amplify exclusionary dynamics or prohibit the transparency open science aims to promote. This commentary paper describes how the unfolding integration of AI into research practices intersects with the goals and challenges of open science, particularly in relation to equity and inclusion. I surface the tensions between open science and research equity, examining how the changing technological landscapes intersect with long-standing socioeconomic and structural barriers. The paper argues for renewed attention to the normative foundations of open science, particularly regarding intellectual property, inclusive infrastructure, and equitable participation.

## Introduction

Science has always relied on epistemological openness to function effectively. The tradition of peer review, the dissemination of research findings, and the collaborative nature of scientific inquiry all rest on principles of transparency and accountability. This openness allows scientific claims to be scrutinised, replicated, and refined by the broader community, facilitating the testing and refinement of empirical knowledge. Openness in science should be understood as both a technical standard and as a principle of just and inclusive knowledge production. Scientific openness also fosters collective progress by enabling researchers to build on one another’s work. In this sense, openness is not an optional value but a foundational condition for scientific credibility, cumulative knowledge, and public trust. However, the historical trend towards openness in science is not linear, and scientific institutions and communities have often been or become ‘closed’ in their practice [1, 2].

Possibilities for inclusive science continue to evolve. The world is becoming more networked, offering greater coordination of infrastructure (institutional repositories, digital object identifiers (DOIs), Open Researcher and Contributor ID (ORCID) accounts, metadata). Training in open scientific practices is becoming more common, increasing open access publication rates, sharing of open source code, and open data. Open Science is supported by many significant policies including the UNESCO Recommendation on Open Science [3]; Plan S [4]; the European Open Science Cloud (EOSC) [5]; the San Francisco Declaration on Research Assessment [6] and, recently, the UNESCO Dubai declaration on open educational resources as digital public goods [7]. Such initiatives anticipate the implementation of an entire open science ecosystem in which the values of equity, accountability, and participation are embedded at the core of research systems.

UNESCO [3] recognises that realising open science means navigating complex and interconnected political, environmental and socio-economic challenges. The benefits of open science—and the ability to participate—are not equally distributed. A recent scoping review [8] found that greater openness does not itself result in increased capacity or inclusion since the capacity to participate in open science in fact varies greatly by geography, demographics, economic advantage and institutional culture. There is an evident tension between the normative aspirations of open science and the structural inequalities that persist across scientific communities. Against this backdrop, the recent and dizzying rise of AI—and particularly GenAI—technologies has added divisiveness and complexity [9]. This commentary paper considers how the unfolding integration of AI into research practices intersects with the goals and challenges of open science, particularly in relation to equity and inclusion.

## Main text

### Open science as democratisation of scientific process

Open science is often framed as a means to democratise the scientific process by expanding who can access, participate in, and benefit from research. Open access publishing allows research outputs to be read by anyone with an internet connection, helping to bridge knowledge divides. Open data and open code enable the reanalysis, replication, and creative reuse of research materials, thereby decentralising expertise and allowing for new insights and applications. Open peer review and participatory research methods, including citizen science [9], offer pathways for non-traditional contributors to influence scientific agendas and validate knowledge claims. Machine learning can improve accessibility and universal design.

From the perspective of epistemic democratisation, there is an opacity and concentration of power inherent in many AI systems. At the point of use, GenAI services are still rapidly proliferating. However, most advanced AI models are developed by a small number of well-resourced corporations and institutions, often using proprietary data, closed algorithms, and resource intensive infrastructure that are inaccessible to most researchers (particularly those in the Global South) [10]. Access to cutting-edge tools and the ability to shape research agendas are thus increasingly centralised by AI in contrast to the open science vision of democratised science. The extent to which these services are truly supporting equitable practice remains debated [11] and how AI companies exploit data uploaded to their platforms is unclear.

The behaviour of AI companies is driven by strategic attempts to garner market share and may embody forms of techno-colonialism [12]. However, AI technologies could be developed and implemented in other ways. Alternative AI models that prioritise transparency, inclusivity, and community governance could offer a more democratic approach to technological development [13]. Open-source and community-led AI initiatives align more closely with the values of openness and epistemic justice. Such models promote transparency by providing access to training data sources, model architectures, and development processes, allowing for scrutiny and reproducibility [14]. Such approaches could foster participation in open science by welcoming contributions from a diverse global community, including researchers from underrepresented regions, interdisciplinary teams, and civil society actors. Some projects foreground ethical considerations, such as multilingual representation, bias mitigation, and, crucially, equitable access to computational resources. BLOOM, for example, was developed through a collaborative, international process directly designed to challenge the dominance of Anglophonic and Western-centric AI models [15]. However, such services are not the norm.

### AI, open science and research integrity

Transparency and reproducibility of research is a cornerstone of scientific integrity [16, 17]. Peer review has traditionally functioned as a safeguard against misinformation and poor methodology, though there are challenges for the traditional model [18, 19]. One of the most pressing challenges facing scholarly publishing today is the exponential increase in research article submissions, raising concerns about research quality, sustainability, and integrity. The ongoing shift from subscription-based access to article processing charges (APCs) in open access publishing incentivises publishers to explore business models. Journals and platforms reliant on APCs often benefit financially from higher volumes of accepted papers. This puts pressure on the research infrastructure. While GenAI has been highlighted as a possible efficiency for peer review [20] GenAI tools are making it increasingly easy for bad actors to quickly create content, shape it into a reasonable approximation of an academic contribution and send it for peer review, exacerbating the problem and resulting in the retraction of published papers [21] and undermining trust.

Academic publishers continue to aggressively defend their market dominance while open alternatives continue to perpetuate [22, 23]. While academic publishing remains highly profitable, the academics and other researchers who contribute intellectual property and labour rarely see such benefits. APCs are also fundamentally inequitable since they discriminate against those with less resources. There is ongoing debate about the move towards more equitable models, such as Diamond Open Access [24] which would improve integrity by mitigating commercial interest.

Interdisciplinary collaboration is particularly critical in AI-driven research. Many of the most pressing scientific questions today require expertise from multiple domains: biologists working with data scientists, engineers collaborating with climate researchers, policymakers engaging with social scientists, and so on. Open science practices make such collaborations more feasible by ensuring that data, methodologies, and findings are freely available for collective analysis. AI tools can also provide ways to coordinate and integrate multidisciplinary research, though they also raise possible questions around the effect on research integrity, such as a lack of authorial oversight when using writing tools or summarising domain specific content.

While open science is often praised for fostering data transparency, sharing, and reuse, Leonelli [25] warns that a simplistic focus on populating a universal commons can inadvertently reinforce epistemic injustice, marginalising diverse research cultures and undervaluing non-mainstream methods. She argues instead for conceptualising open science as “judicious connection” between diverse systems of practice and research cultures. Such approaches emphasize ongoing reflection, contextualisation, and community-building rather than simply relying on open access publication as a sufficient condition for open science.

Open-access publishing plays a crucial role in broadening knowledge dissemination, making research findings available to a wider audience. AI tools that purport to help researchers navigate the vast amount of literature being produced such as search engines and literature summary/synthesis tools can potentially play an important role, but greater attention needs to be paid to their effects on the integrity of research practices [26].

### Open science for equity and inclusion

The call for “judicious connection” [25] in open science is also relevant across issues of equity, diversity and inclusion. Science and academia remain fundamentally inequitable, dominated by wealthier institutions from the Global North. Historically, access to scientific literature has been restricted by paywalls, APCs, institutional limitations, and opaque data-sharing practices. English has become the de facto language of global science, privileging native speakers and creating barriers for researchers working in underrepresented or low-resource languages. Linguistic prevalence limits the visibility and perceived legitimacy of non-English research, marginalising localised knowledge and reinforcing patterns of epistemic exclusion. Researchers who must translate or adapt their work for international publication face additional labour and cost burdens, which further exacerbate inequities in dissemination and recognition.

Although open science encourages data sharing, preprints, and collaborative methods, these practices often go unrewarded in career evaluations that continue to privilege high-impact journal publications. These barriers disproportionately impact researchers in low- and middle-income countries, independent scholars, and those outside traditional academic institutions [27, 28]. While the platformization of citizen science [9, 29] has expanded opportunities for public participation in scientific research, such opportunities are not equally distributed.

Can AI support greater equity, diversity and inclusion (EDI) in open science? The answer depends less on the technology itself and more on how it is designed, governed, and integrated into research ecosystems. AI has the technological potential to increase access to knowledge, reduce technical burdens, and enable new forms of participation. AI-assisted translation and language refinement tools could enhance accessibility, allowing researchers to communicate their findings across linguistic and cultural boundaries with greater ease, offering the possibility of a meaningful step toward a more multilingual and inclusive open science ecosystem. However, EDI considerations are often neglected in system development [30] and are often reduced to matters of technical accessibility rather than socio-economic or political considerations [31]. This has led to calls for more socially responsible system design [32] and a need to address structural barriers more directly [33].

There is a growing recognition of the need to balance global alignment with local sensitivity [25]. Open science must be adaptable to diverse epistemologies, knowledge systems, and community needs. An illustrative challenge is exemplified by the tensions between the imperative to share knowledge as widely as possible coming into potential conflict with traditional or indigenous knowledge systems which may have their own protocols for sharing [34, 35]. In such cases, EDI is respected by refraining from the attempt to make all forms of knowledge as widely available as possible.

## Outlook

Openness is a fundamental principle that underpins the scientific ecosystem. By strengthening transparency, accessibility, and impact, open science enhances the methodological, epistemological and ethical dimensions of research. In the age of AI, maintaining openness is more crucial than ever for ensuring rigor, reproducibility, and public trust. The outlook for open science within the global research community is one of both growing momentum and increasing complexity. Legal frameworks have not kept pace with technological progress [7] which means that members of research ecosystems must ethically reflect on their practice and their contributions to open science.

There is an ongoing need for knowledge transfer services that can support discourses and make participation possible for a wider range of stakeholders. AI can potentially build capacity that supports equitable participation [8] but there are significant challenges in avoiding algorithmic discrimination [36]. There is also heightened awareness of the risks posed by unrestricted access to increasing amounts of data, including data misuse, and issues of research security. As a result, open science is evolving to a more nuanced set of practices with complex legal, ethical and epistemological obligations. The challenge ahead lies in developing inclusive, interoperable frameworks that support global collaboration while safeguarding research integrity, privacy, and trust; and expanding EDI through addressing structural as well as technical barriers to participation. International collaboration is essential, but this must be equitable and not just a way for Global North actors to export unethical practices [37] under the banner of being “open”.

## Data Availability

No datasets were generated or analysed during the current study.

## Abbreviations

APCs: Article Processing Charges
AI: Artificial Intelligence
DOIs: Digital Object Identifiers
EDI: Equity, Diversity and Inclusion
EOSC: European Open Science Cloud
GenAI: Generative Artificial Intelligence
ORCID: Open Researcher and Contributor ID
